# The complete mitochondrial genome of *Antheraea proylei* strain In981 (Lepidoptera: Saturniidae)

**DOI:** 10.1080/23802359.2019.1627944

**Published:** 2019-07-12

**Authors:** Jian Yang, Ru-Song Zhang, Dong-Bin Chen, Miao-Miao Chen, Yu-Ping Li, Yan-Qun Liu

**Affiliations:** a Department of Sericulture, College of Bioscience and Biotechnology, Shenyang Agricultural University, Shenyang, China;; b Sericultural Institute of Liaoning Province, Fengcheng, China

**Keywords:** *Antheraea proylei* In981, mitochondrial genome, phylogenetic relationship

## Abstract

In the present study, we report the complete mitochondrial genome of *Antheraea proylei* strain In981, a hybrid of Chinese oak silkmoth (*A. pernyi*) and Indian oak silkmoth (*A. roylei*). The circular molecule is 15,573 bp in length, with 37 typical coding genes (13 protein-coding genes, 2 ribosomal RNA genes, and 22 transfer RNA genes) and one non-coding A + T-rich region of 552 bp long. Its gene components and gene order are identical to the common type found in Bombycoidea species. Phylogenetic analyses revealed that In981 is closely related to *A. pernyi* rather than *A. roylei*. This is the first report on the complete mitochondrial genome of *A. proylei*.

Indian oak silkworm *Antheraea proylei* is a synthetic hybrid derived from the fertile hybrid of *A. roylei* of India and its Chinese counterpart *A. pernyi* (Nagaraju and Jolly 1986). The interspecific hybrid developed for tasar silk produced in India has been introduced into China in 1998 (Wang et al. 2002). However, the molecular information regarding this economically important silkmoth remains severely limited, and only a handful of DNA sequences are available. In this present study, the complete michondrial genome of this species was determined for the first time, providing a basic genetic information for this hybrid.

The inbred strain In981 of *A. proylei* has been successively kept at the Sericultural Institute of Liaoning Province (N40°28′16.45′′; E124°04′20.30′′), Fengcheng, China. A single larva was used to extract the total DNA and stored at our lab. Two overlapping fragments of ∼8 kb were amplified with specific primers. Then, the PCR amplifications were purified and sequenced on Illumina Hiseq platform by Frasergen Co., Ltd., Wuhan, China. A reference-guided assembly was used to reconstruct the mitochondrial genome, and *A. pernyi* (AY242996; Liu et al. 2008) served as the reference. The genome was annotated with MITOS (Bernt et al. 2013) and manually corrected based on the known mitochondrial genomes of *Antheraea* species. The mitochondrial genome of *A. proylei* In981 has been deposited in GenBank under the accession no. MK920216.

The mitochondrial genome of In981 is 15,573 bp in length and contains a typical gene complement of metazoan: 13 protein-coding genes (PCGs), 22 tRNA genes, 2 rRNA genes, and an A + T-rich region. The order and arrangement of this genome is identical to those of Saturniidae species available. All protein-coding genes start with a typical ATN initiation codon, except for *COI* that begins with atypical codon CGA. Nine of 13 PCGs have a complete termination codon (TAA or TAG), but *COI*, *COII*, *ND5*, and *ATP6* terminate with the incomplete stop codons T–– or TA–. The A + T-rich region of In981 spans 552 bp long and harbors a repeat region composed of six 38 bp tandem repeat units, as found in *A. proylei* (DQ415454) and four *A. pernyi* strains (AY242996, KP762788, KP881616, and KP999979). In contrast, *A. roylei* contains five 38 bp tandem repeat units in the A + T-rich region (Arunkumar et al. 2006).

We built the phylogenetic relationship using Bayesian inference in Mrbayes 3.1.2 (Huelsenbeck and Ronquist 2001). The phylogenetic tree based on partial *COI* sequence corresponding DNA barcoding confirmed that In981 belongs to *A. proylei* and very close to the domestic type of *A. pernyi* followed by the wild type of *A. pernyi* (Liu et al. 2012) and *A. roylei* (Figure 1(A)). The whole mitochondrial genome further provided a relationship that In981 and the the domestic type of *A. pernyi* were closely related (Figure 1(B)). More samples from *A. proylei* individuals and *A. roylei* are needed to be sequenced to understand the paternal inheritance of mitochondrial DNA of this hybrid (Arunkumar et al. 2006).

**Figure 1.**
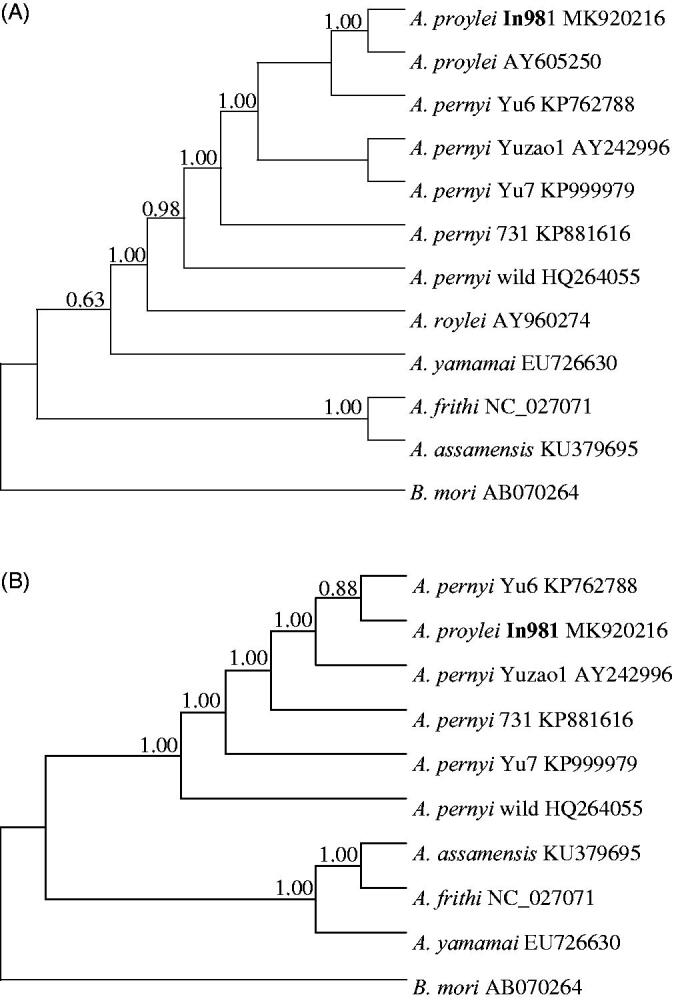
Phylogenetic relationships among *Antheraea* species inferred from the partial *COI* gene sequence (A) and whole mitochondrial genome sequence (B) using Bayesian inference with GTR + G + I model. *Bombyx mori* serves as outgroup. The posterior probability values are indicated at the nodes. GenBank accession numbers are listed following the name of each species or strain.
